# Science and policy to manage nitrogen with cover crops—The Maryland experience

**DOI:** 10.1002/jeq2.70316

**Published:** 2026-10-27

**Authors:** Ray Weil, Alisha Mulkey

**Affiliations:** ^1^ Department of Environmental Science and Technology University of Maryland College Park Maryland USA; ^2^ Maryland Department of Agriculture Annapolis Maryland USA

## Abstract

During the 1980s, research showed nitrogen‐stimulated eutrophication was contributing to the Chesapeake Bay's deterioration, and cover crop research shifted from legumes to replace fertilizer, to winter cereals to reduce nitrogen (N) leaching. The Maryland Department of Agriculture (MDA) adopted nutrient regulations and added technical and financial resources promoting N‐capturing cover crops and N‐use efficiency. Today, Maryland's “Clean the Bay” effort includes regulations and generous cost‐sharing for planting and terminating cover crops. The MDA supports cover cropping on ∼180,000 ha annually, out of ∼350,000 ha of land in row crops not planted to winter cereals, achieving by far the nation's highest percentage (∼50%) of cropland in cover crops. Brassica species gained popularity because they effectively capture N when planted early, and also provide immediate farmer benefits, such as alleviating soil compaction, suppressing certain weeds, and avoiding N immobilization. The MDA tweaked its program to include brassicas and, later on, legumes in mixtures with grasses. Still, research showed large profile N pools remaining in fall, much of it deeper than 90 cm. Further work indicated this residual N could be cleaned up by early‐planted, deep‐rooting cover crops. Additional progress on enhanced cover cropping included inter‐seeding to start growth early, multiple species for stand reliability and immobilization avoidance, and planting “green” into living cover crops for maximizing soil health and water conservation benefits. Maryland's Cover Crop Program is modified annually, with recent changes incentivizing more time for cover crops to function at each end of their growing season.

## Plain Language Summary

We discuss 50 years of nitrogen (N) management in the Chesapeake Bay watershed, focusing on cover crops. During the 1980s, research showed N‐stimulated eutrophication was a major cause of the Bay's water quality deterioration. Soon, cover crop research shifted focus from legumes to replace fertilizer, to winter cereals to reduce N leaching. The Maryland Department of Agriculture (MDA) adopted nutrient regulations along with technical and financial resources promoting N‐capturing cover crops and N‐use efficiency. Today, Maryland's “Clean the Bay” effort includes up to 100% cost‐sharing for planting and terminating cover crops on ∼180,000 ha annually, out of the state's ∼350,000 ha of land in row crops not planted to winter cereals. The MDA has tweaked its program to incentivize brassicas, legumes in mixtures with grasses, early fall planting, and late spring termination to maximize cover crop effectiveness in capturing N and building soil health.

**Abbreviations**
MDA: Maryland Department of Agriculture
PSNT: pre‐sidedress nitrate test
TMDL: total maximum daily load

## 1 BACKGROUND ON MARYLAND AGRICULTURE AND THE CHESAPEAKE BAY

The US state of Maryland is home to one of the world's great estuaries, the Chesapeake Bay. During the late 19th and early 20th centuries, the Chesapeake Bay was highly productive, and Maryland was the world capital of oyster production (Schulte, 2017). The productivity of the Chesapeake Bay declined precipitously during the 20th century due to a combination of overfishing, exotic diseases, and environmental pollution of its waters. By the early 1980s, the Chesapeake Bay was declared nearly dead. Investigations into the causes of its declining water quality implicated three major water pollutants: nitrogen (N), phosphorus (P), and sediment. During the summers, eutrophication was severe, and sediment caused such turbidity that underwater grasses, which serve as fish breeding grounds, severely declined.

During the 1980s, a multistate, multi‐partner effort began to restore the Chesapeake Bay with a regional U.S. Environmental Protection Agency program developed to reduce nutrient and sediment loading. Nitrogen‐stimulated eutrophication was identified as a major cause of the Chesapeake Bay's deterioration, especially in the southern, more brackish portion of the Chesapeake Bay. Effort and funding were directed toward reducing the N loading coming from inefficient water treatment plants and N‐fertilized agricultural fields. The main agricultural products produced on Maryland cropland are corn, soybeans, wheat (and other small grains), vegetables, and fruits. Dairy production is common on the Western shore of the Bay, while broiler production is widespread on the lower Eastern Shore.

On poultry farms on the Eastern Shore of the Chesapeake and dairy farms on the Western Shore of the Chesapeake, a lack of analytical resources and education on manure nutrient value resulted in rather uninformed management of manure application rates prior to the 1990s. Farmers rarely based application rates on the nutrient analysis of manure samples. Lacking manure analysis information, they were not confident about how much N the manure would actually supply, and therefore, they would typically apply 100+ kg ha^−1^ of fertilizer N *along with* rates of poultry litter in the range of 15–30 Mg ha^−1^. During this period, nitrate was found in high levels (20–30+ mg N/L) in groundwater under Eastern Shore cropland during the winter and spring, especially where poultry manure was spread, even when corn yields were high (Weil et al., 1990).

## 2 EARLY STEPS IN NITROGEN MANAGEMENT

Since information on manure nutrient content and N availability would facilitate the management of manure as a nutrient resource, one of the first steps in the nutrient management effort that the State of Maryland instituted was to offer farmers free manure sample analysis through the University of Maryland Soil Testing Laboratory. Armed with manure analysis data and research (Hadas & Portnoy, 1994; Preusch et al., 2002) on rates of N release from manures, it was soon determined that typical poultry manure application rates during that period supplied far more N (and P) than needed for optimal crop production. The new analysis‐based recommendations for poultry manure application rates were in the range of 1–5 Mg ha^−1^ (a quarter as much as was previously common). These analysis‐based rates were so much lower than those previously used that the existing manure‐spreading equipment could not evenly apply such small amounts. New spinner‐spreaders were designed and have since become standard for the uniform application of appropriate rates of poultry litter. Similar advances were adopted in the technology for spreading dairy manure at appropriate rates, mainly involving liquid manure spreaders and injectors (Hansen, 2006).

Other practices were also promoted to improve N‐use efficiency and minimize surplus N susceptible to leaching loss. These practices included basing N application rates on realistic yield goals that reflect actual yields achieved in recent years, rather than wishful thinking or rumors of the neighbors’ yields. Another practice recommended was the split application of N fertilizer. With this approach, N fertilization was split into two applications: a small planting‐time application near the seed row, and an application of most of the N as a sidedress in June when corn is in the V4–7 stage. The split application strategy prevents most of the soluble N from exposure to leaching and volatilization processes during the 6–8‐week period between corn planting and the onset of rapid N uptake. It also allows for more flexibility in determining an N application rate that considers early‐season weather and soil conditions. Nitrogen efficiency using urea and urea‐ammonium nitrate solutions was observed to be limited by rapid urease reactions in the no‐till surface residue layer and subsequent ammonia volatilization. Nitrogen efficiency was enhanced by replacing a surface spray with knife‐injection and dribbling technologies (Lamond et al., 1991) that ensured that most of the liquid fertilizer was placed below the soil surface, thus substantially reducing ammonia volatilization (but possibly increasing nitrate leaching potential).

No‐till soil management was researched and promoted in Maryland during the 1970s–1980s. By the mid‐1980s, reduced tillage and no‐till farming were widely adopted in Maryland. Early work by Allen Bandel and others (Bandel & Fox, 1984; Bandel et al., 1975) compared no‐till to conventionally tilled corn. Their work suggested that as surface soil organic matter built up under no‐till, especially when winter cereal cover crops were included, the microbial decomposition of residues competed with crops for N, requiring the application of 30–40 kg ha^−1^ of extra N to prevent a corn yield drag during the first few years of no‐till management. Lessons about N immobilization, similar to those learned in the 1980s, continue to be relearned elsewhere (Gentry et al., 2024; Quinn et al., 2021, 2023; Ruffatti et al., 2019; Waring et al., 2020).

Despite all these efforts, research at the University of Maryland (Magette et al., 1989) reported that even with all the best management practices implemented, N was likely to leak from cropping systems on permeable coastal plain soils to an extent that could drive eutrophication problems and even exceed drinking water nitrate limits in groundwater. They concluded that “current best management practices are not capable of allowing producers in coastal plain regions to always comply with a nitrate standard for groundwater of 10 mg/L of nitrate‐nitrogen.”

During the 1990s, the University of Maryland, in conjunction with the Maryland Department of Agriculture (MDA), developed an initially voluntary Nutrient Management Program to help farmers address concerns about excessive N and P loading to the Bay (Blankenship, 1994). Although these two eutrophication‐causing nutrients are both intimately associated with the management of organic wastes, here we will consider only the N part of the story. Nitrogen is often the limiting factor determining crop yields, but its availability during any particular crop growing season is difficult to predict. Therefore, farmers in Maryland, as elsewhere, tended to over‐apply N on average as they sought to position their crops to take advantage of especially favorable growing conditions that might occur in any given year.

In the 1980s, to better assess the need for N fertilizer in a growing season, Fred Magdoff of the University of Vermont developed an in‐field incubation procedure that came to be known as the pre‐sidedress nitrate test (PSNT) (Magdoff et al., 1984). Researchers at the USDA and the University of Maryland worked to adapt this N test to Maryland conditions in the early 1990s (Meisinger et al., 1992). This test was based on 30‐cm deep soil samples obtained in June, when corn is at the V5 stage of growth. The soil nitrate concentration at this time was found to be a good indication of how rapidly N was mineralizing from soil organic matter, cover crop residues, and/or previously applied manure, as well as how much N may have been lost by leaching or denitrification by that time due to rainy weather and wet soils. Thus, waiting until sidedress time to apply the bulk of the N allowed a better assessment of the N‐supplying capacity of the soil for that particular season. The PSNT is still widely used in Maryland and the mid‐Atlantic region, with the critical nitrate concentrations obtained by the PSNT recently reevaluated for use in modern high‐yield corn systems (White et al., 2023).

## 3 CONTINUED TECHNICAL AND POLICY PROGRESS

In 2010, the EPA, under its Clean Water Act authority, issued a total maximum daily load (TMDL) to address the persistently impaired waters. The Chesapeake Bay TMDL imposes maximum loading limits for N, P, and sediment, whereby each Bay watershed jurisdiction is allocated a portion of the maximum pollutant load relative to each jurisdiction's contribution to the Bay watershed flow. Jurisdictions are operating under their Phase 3 Watershed Implementation Plans, submitted to EPA in 2018, to outline the programs and strategies each will employ to achieve the prescribed nutrient and sediment reductions in the Bay TMDL. Cover crops are a key strategy identified in Maryland's Watershed Implementation Plan (Maryland Department of Environment, 2025).

There is now detailed county‐by‐county evidence for the Chesapeake Bay region that the inputs of N to agricultural land, and even more so for P, trended downward, and that N and P use efficiency has trended upward from 1990 to 2020 in the region (Zou et al., 2024). In addition to the already‐discussed N management measures, basing N rate recommendations on a quadratic‐plateau approach to N response data rather than assuming a simple quadratic response curve may have resulted in reduced N application recommendations (Morris et al., 2017). Other avenues used to reduce N applications included using precision agriculture variable‐rate N application equipment with N rates based on spatial yield zones mapped from previous years' harvest data or on normalized difference vegetation index sensing during the N application operation (Scott et al., 2022).

Data available for the sales of N fertilizer and corn production in Maryland during the first three decades of the Maryland Nutrient Management Program suggest similar and possibly even more pronounced trends in the State of Maryland (Figure 1). Also shown in Figure 1 are calculated average N fertilizer application rates for corn and the average applied N‐to‐dry grain production ratio. Average rates of N fertilization declined in Maryland, even while the average corn yields increased. Corn yields in Maryland paralleled those in the US corn belt for the same period. Before the full implementation of the MDA nutrient management program, apparent N‐use efficiency was targeted at about 0.017 kg N applied per kg of corn grain produced (1 kg of N for each 58 kg of corn grain yield, known colloquially as the “one pound N per bushel of expected yield” rule of thumb). From 1992 to 2022, the kg of N fertilizer used to produce 1 kg of corn grain in Maryland dropped from an average of nearly 0.014 to about 0.008 (Figure 1). It should be noted that the calculations behind Figure 1 are approximate because corn uses most, but not all, of the N fertilizer sold in Maryland, and N fertilizer is supplemented on some farms with poultry litter or dairy manure.

**FIGURE 1:**
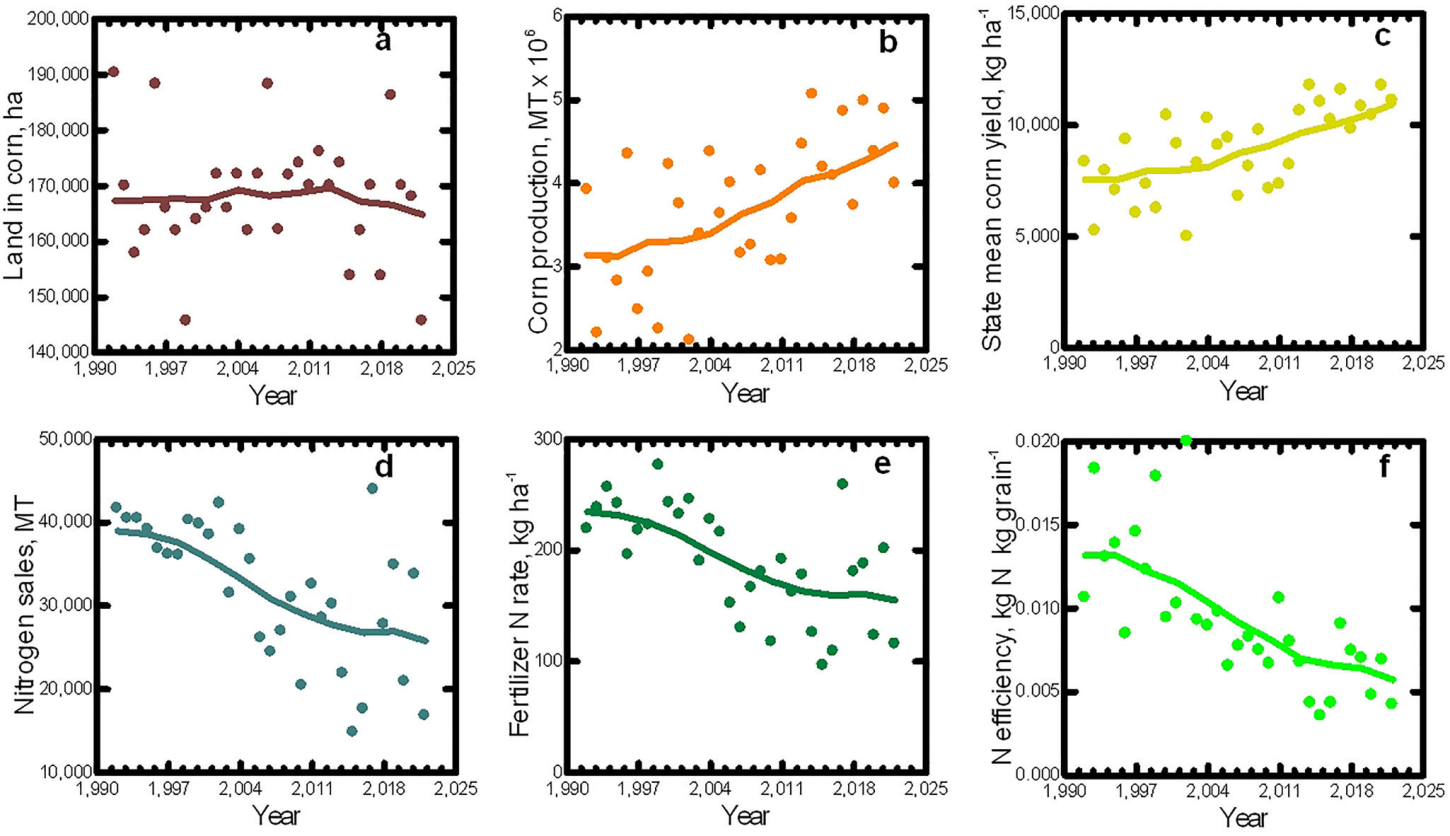
Trends in corn production and N fertilizer use in Maryland during three decades under the Maryland Nutrient Management Program.(a) Land in corn production, (b) corn production per year, (c) mean corn yield per hectare (panel [b] data divided by panel [a] data), (d) nitrogen sales per year, (e) fertilizer N sales per area of corn production (panel [d] data divided panel [a] data), and (f) fertilizer N use per kg of corn produced (panel [e] data divided by panel [c] data). Average corn yields went up at about the same rate as seen in the US corn belt for the same period, but rates of N fertilization went down. The kg of N fertilizer required to produce 1 kg of corn grain in Maryland dropped from nearly 0.014 to 0.008. The calculated variables do not account for the facts that corn uses the vast majority, but not all, of the N fertilizer sold in Maryland, and that N fertilizer is supplemented on some farms with poultry litter or dairy manure. These two factors have not changed significantly during the period. Data on N fertilizer sales from the Maryland State Chemist (personal communication, 2022); other data from the US Department of Agriculture National Agricultural Statistics Service (USDA/NASS, 2024).

Even with the adoption of more conservative N application rates, reductions in N loading to the Chesapeake Bay were smaller and slower than expected. During the 1980s, University of Maryland researcher Russel Brinsfield established adjacent comparison watersheds at the Wye Research and Education Center situated on the Eastern Shore of the Bay. The side‐by‐side watersheds were instrumented so that hydrology and nutrient flows in both surface runoff and groundwater could be monitored. The initial objective was to compare no‐till agriculture to conventional tillage. During the late 1980s and early 1990s, researchers (Staver & Brinsfield, 1998) added a cereal rye cover crop to the system in both watersheds.  With careful monitoring of groundwater over time, this research determined that including a cereal rye cover crop after corn dramatically reduced nitrate leaching within just a few years. Nitrate‐N concentrations in groundwater fell from 15–25 to <5 mg L^−1^ within 6 years for the no‐till system and within 9 years for the conventional till system. Cover crops seemed to be able to dramatically reduce the nitrate leaching problem that earlier work suggested was an inevitable consequence of high‐yield crop production. These data were so convincing that the MDA and University of Maryland Extension began to promote the planting of rye and other winter cereal cover crops to scavenge leftover soil N and reduce the leaching of nitrate.

During the unusually hot summer of 1997, harmful algal blooms of *Pfiesteria piscicida*, a toxic dinoflagellate, occurred in the lower Chesapeake Bay, presumably abetted by high N and P levels in the estuary. The Pfiesteria resulted in fish kills and aerosol‐borne human health effects (Swinker et al., 2002). With the ensuing “*Pfiesteria hysteria*” among the public, Maryland politicians sought to transform the previously voluntary nutrient management program into a mandatory program of N load reduction with regulations, not just recommendations (Terlizzi, 2006). A new source of tax revenue was legislated (the so‐called “flush tax”) to support various approaches to reduce N loading to the Chesapeake Bay. This new tax was featured quite prominently on Maryland residents’ quarterly water bills (Figure 2). As compared to $35–$400+ per kg of N load prevented by urban stormwater management and water treatment plant improvements, winter cereal cover crops were seen as a cost‐effective measure to reduce N leaching from cropland, costing as little as $10 per kg of N load prevented (Brainard, 2014). Based on an analysis of costs and benefits, the Chesapeake Bay Program set a goal for Maryland to reduce the annual N loading to the Bay by some 9 million kg year^−1^. About 40% of the reduction was to be addressed by water treatment plant upgrades and other point‐source investments, while the remaining 5 million kg of N year^−1^ was to be achieved by adoption of agricultural best management practices (Blankenship, 2002). These coordinated N reduction efforts, which included cover cropping, have indeed reduced eutrophication. A recent analysis suggests that the N loading reductions achieved between 1985 and 2020 reduced the summer hypoxic (O_2_ < 1 mg L^−1^) water volumes in the Bay by 20%–70% from what they would have been without the N loading reductions, even with warming trends working in the opposite direction (Frankel et al., 2022).

**FIGURE 2:**
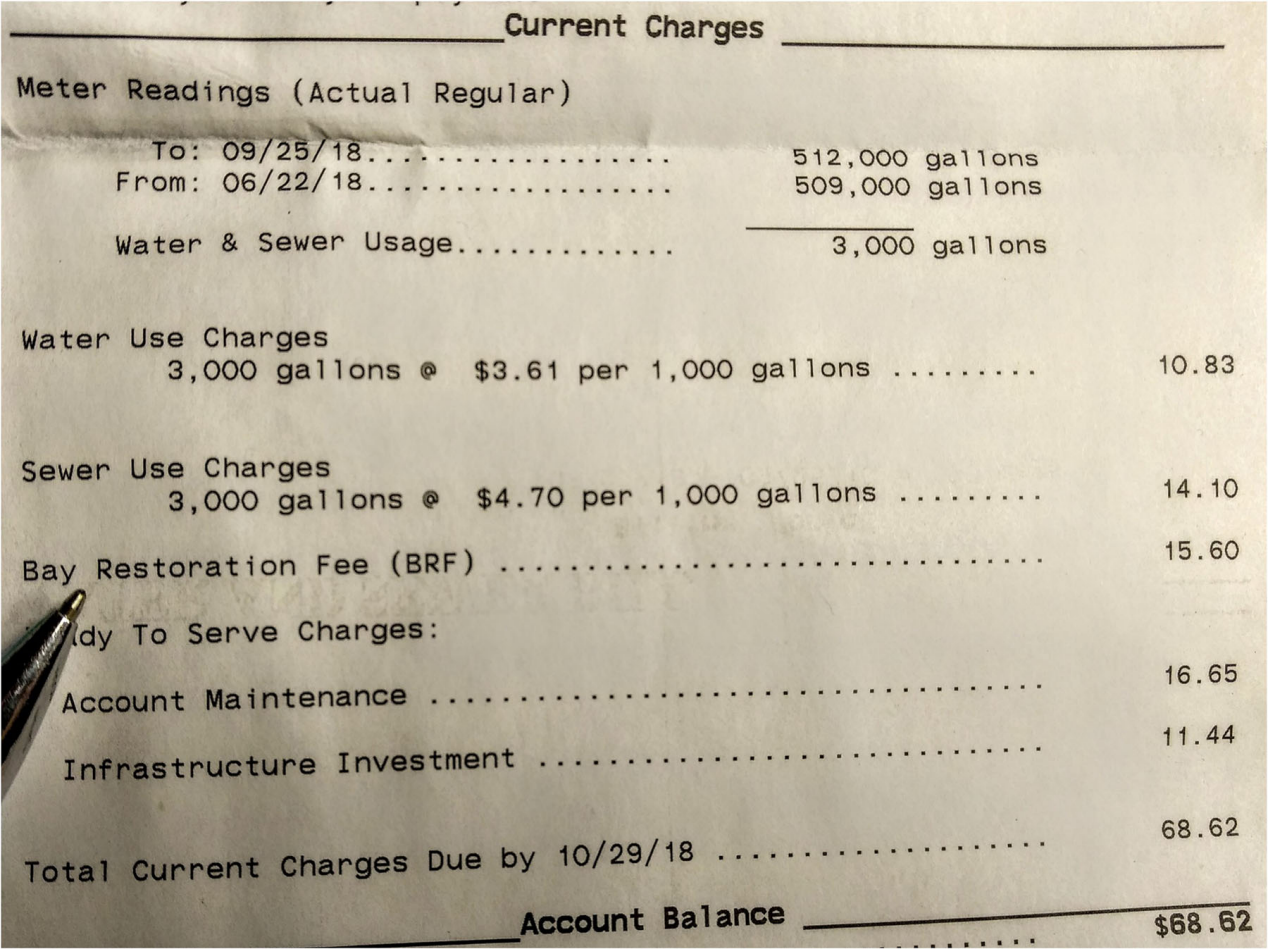
Since the early 2000s, the Maryland “flush tax” or Bay Restoration Fee has been featured prominently on every consumer's water/sewage bill. Photo by R. Weil.

## 4 COVER CROP PROMOTION AND COST‐SHARE

By the turn of the 20th century, cover crops were widely promoted by the University of Maryland Extension, and the MDA initiated a program to subsidize the use of cover crops. Initially, in the late 1990s, the subsidy or “cost‐share” averaged about $65 ha^−1^, and farmers enrolled about 145–165,000 ha in the program. To meet the target of growing cover crops on 185,000 of Maryland's 400,000 ha of cropland, the base subsidy levels were increased in 2004 to an average of $85 ha^−1^ and then in 2010 to over $110 ha^−1^. Also, in 2010, limits were removed for how many hectares a farm could enroll and the total payment a farm could receive. In addition, the Maryland cover crop program offered extra add‐on subsidies targeted at practices that research suggested would enhance the reductions in nitrate leaching achieved by cover cropping. Programmatically, incentive levels are based on available program budgets each year, and the prioritization of incentives (e.g., planting date, planting method, etc.) is set, foremost, for water quality, with increasing recognition of the soil health and climate adaptation benefits of cover crops.

Maryland farmers generally responded positively to these subsidies, which were significantly higher than those offered in other states. Since 2010, the State of Maryland's enrollment target of 185,000 ha of cover crops has generally been achieved and often exceeded (Figure 3). This level of adoption out of the approximately 350,000 ha of Maryland land in row crops not planted to winter cereals (USDA/NASS, 2024) achieved by far the nation's highest percentage (∼50%) of cropland in cover crops. The high cover crop adoption rate for Maryland cropland is at least partially due to the flexible and responsive nature of the MDA cover crop subsidy program. The MDA constantly tweaks the incentive structure and rules for the cover crop subsidy program in response to new research findings, farmer practices, policy objectives, and even real‐time weather conditions. Two examples of the Program's responsiveness follow.

**FIGURE 3:**
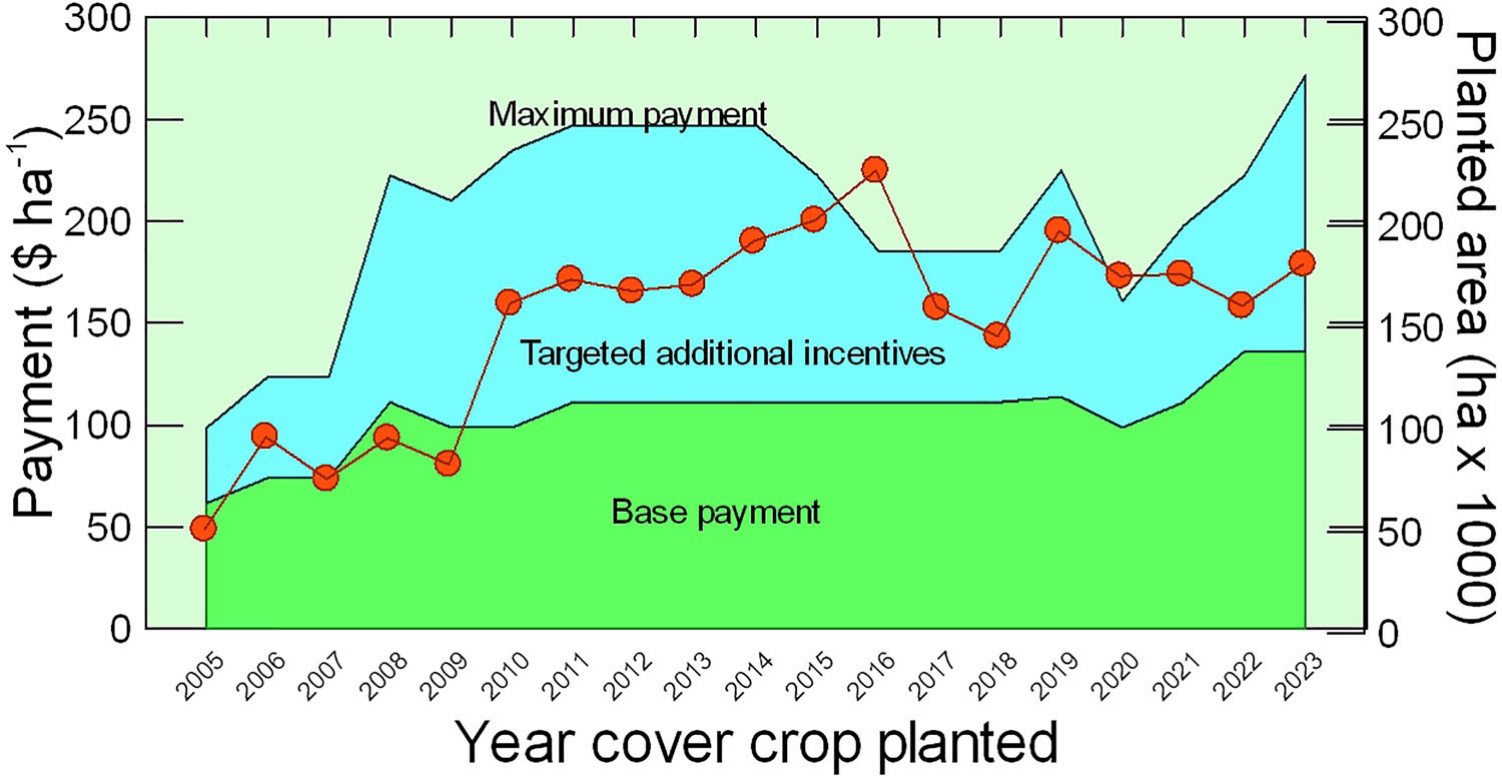
The State of Maryland's base and maximum incentive payment rates and cropland area enrolled in the Maryland Department of Agriculture (MDA) Cover Crop Program from 2005 through 2023. The maximum payment jumped after 2008 when the base payment was increased to ∼$100 ha^−1^. In subsequent years, additional payments over and above the base payment were made to incentivize practices such as planting early, using cereal rye, and cover cropping fields that received manure applications. With higher payments and no acreage caps after 2008, the mean annual land enrollment generally met the State's 180,000 ha target for restoring water quality in the Chesapeake Bay. *Source*: Unpublished data from MDA (Dawn Bradley, personal communication, January 20, 2022).

In the first example, research in the early‐ and mid‐2000s (Dean & Weil, 2009) showed that brassica cover crops could be as or more effective than winter cereals in scavenging N and preventing nitrate leaching. The MDA responded to this new information by including brassicas and mixtures of brassicas and winter cereals in the cover crop subsidy program in 2014. Research also showed that the effectiveness of brassica cover crops in capturing N in falls dropped off radically if they were planted later than mid‐September. The MDA cover crop program instituted a 15 September cutoff date for seeding brassica cover crops to ensure their successful establishment and effective N capture.

A second example involves the inclusion of legume cover crops in the program. Since the primary objective of the cover crop program was to reduce N loading from cropland, legume species were not allowed in program‐subsidized cover crops. It was thought that legumes’ N2‐fixation would add to the N in the system and reduce the N‐capturing activity of the non‐legumes in cover crop mixtures. However, research in the early 2010s showed that legumes in a mixture with brassicas and or winter cereals did *not* reduce the capacity of the non‐legumes to capture N (Hirsh et al., 2021). In addition, the legumes grew little in fall and so would fix N mainly in spring, when that biologically fixed N could reduce the amount of fertilizer N needed. Therefore, the MDA policy was adjusted to allow planting legumes in fall cover crop mixtures starting in 2016.

## 5 EARLY PLANTING TO REDUCE NITRATE LEACHING

In the early 2010s, serendipitous observations of darker green, taller strips of fall‐planted cover crops (Figure 4) led to the hypothesis that cover crops may be capturing N from quite deep in the profile, not just from the topsoil.  The greener strips were observable in certain fields because some farmers applied sidedress N down the middle of every second row of corn, rather than every row, thus providing N to the corn plants on one side or the other. Given the standard 72‐cm row spacing for corn, with this method of sidedressing, the N was applied in strips 154 cm apart. Greener, taller cover crops in strips 154 cm apart observed in November were obviously being stimulated by strips of N applied as sidedressing to corn the previous June. In fields using the more common method of sidedressing N to every corn row, the N bands were too close together to produce distinctly greener, taller plants in the following cover crops. Measurements of cover crop N uptake in the greener and less green strips suggested that about half of the N applied in June had not been used by the corn but was still available to be taken up by cover crops in the months after corn harvest. In addition, measurements made to 1‐m depth showed only about one‐third of the extra N in the cover crops was accounted for by less mineral N remaining in the soil (Wang & Weil, 2018).

**FIGURE 4:**
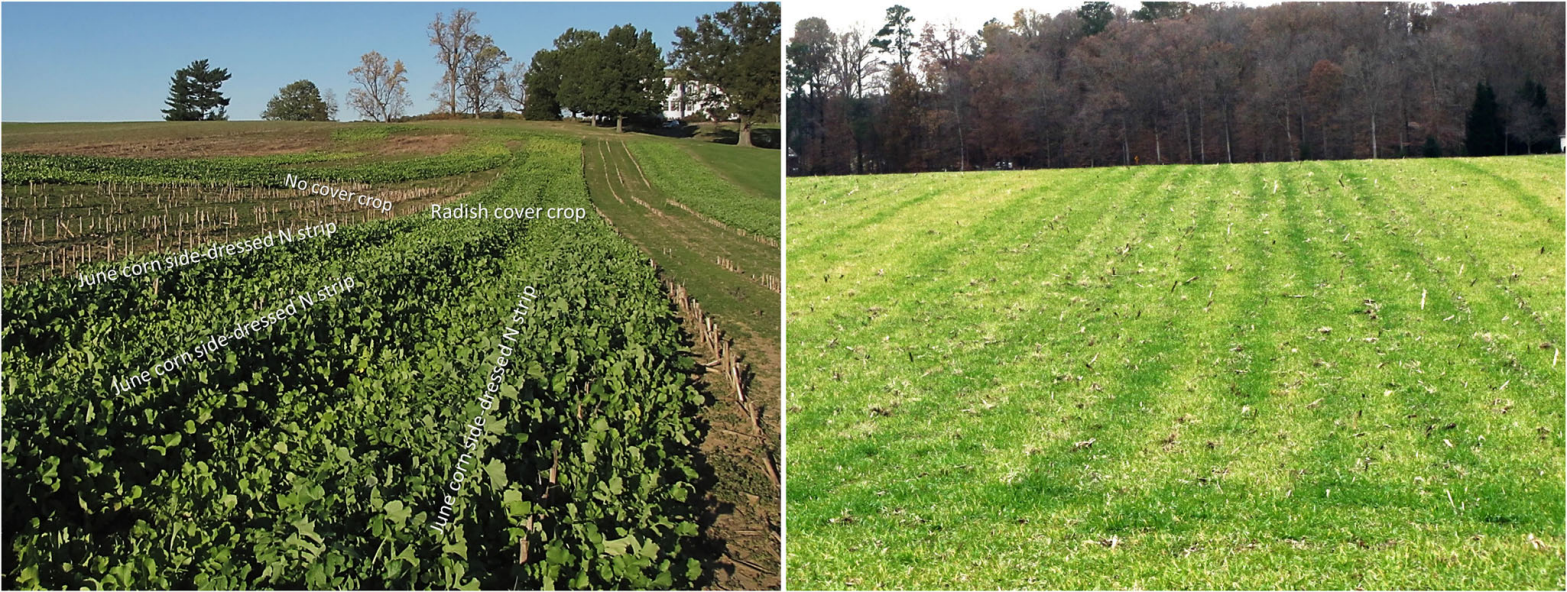
Two examples where sidedressing liquid N fertilizer down the middle of every second corn row in June, resulting in bands of N 154 cm apart. Cover crops (radish on the left, rye on the right) drilled after corn harvest responded to the bands of fertilizer N still available in the soil in late fall. Photo by R. Weil.

One hypothesis generated to explain the above trends was that cover crops were capturing N from deeper than 1 m in the soil. Subsequent research (Hirsh & Weil, 2019) showed that large pools of soluble nitrate and ammonium were present throughout the profile of soils under most crop fields in the mid‐Atlantic region, averaging about 250 kg of mineral N per hectare in the upper 2 m. About half of this soil mineral N was in the form of nitrate, and about half of that was present deeper than 90 cm. They reasoned that soluble N already deeper than 60 cm in the profile at the end of the summer growing season would be especially susceptible to leaching loss to groundwater and from there to streams and the Chesapeake Bay.

Working with stable ^15^N isotope‐labeled nitrate, Hirsh (2018) showed that roots of cover crops planted around September 1 could reach and take up labeled nitrate from a depth of 2 m before freeze‐kill or winter dormancy set in. This observation led to the hypothesis that the role of cover crops in reducing nitrate leaching was largely tied to deep rooting and N uptake in the fall before the cold winter conditions caused cover crop dormancy. The low levels of nitrate in drainage water observed all winter and into early spring were probably not due to continuous uptake of N during the whole period, but rather to the cleaning up of soluble N from the whole profile in the fall, so that drainage water would not encounter much soluble mineral N that it could carry away during the winter and spring. The importance of N deep in the soil profile also suggested that nitrate leaching was not as closely related to fertilization of corn as previously thought. In fact, in the abovementioned survey study (Hirsh & Weil, 2019), more nitrates were found in the deep soil profile under soybean crops that received no N fertilizer than under N‐fertilized corn crops.

Most N leaching in the mid‐Atlantic region occurs as the water tables are rising during the fall–winter–spring months, when evapotranspiration is much lower than precipitation. Thus, the main leaching season spans from the time deciduous trees lose their leaves in October–November through April–May, when deciduous trees leaf out again and begin transpiring water. With this in mind, research showed that early planting of cover crops was necessary to allow them to remove most of the soluble N from the soil profile before the main leaching season. Cover crops planted after corn or soybean harvest might not have sufficient time to clean up the profile before they winterkill or go dormant for the winter. Furthermore, in some situations, planting early enough to achieve this objective might be practical only through interseeding cover crops into standing summer crop canopies before harvest time. Unless irrigation is available, it is challenging to achieve even cover crop stands by broadcasting cover crop seed into standing cash crop canopies (whether by high clearance ground seeders or airplanes). However, research suggests that for reducing nitrate leaching, the extra growing degree days available with early interseeding generally make up for the more even stands achieved by waiting 3–4 weeks to drill the cover crop seeding after cash crop harvest (Sedghi & Weil, 2022; Sedghi et al., 2023). This knowledge is reflected in the Maryland cover crop program adjustments to incentivize early planting of N‐capturing species.

## 6 CONTINUING CHALLENGES FOR NITROGEN MANAGEMENT

Intensive field N‐response research in Pennsylvania resulted in an empirical model (Arrington et al., 2024) that suggests surprisingly little effect of cover crop N content on economic optimum N rate for a following corn crop. Conventional wisdom and academic meta‐analyses (Marcillo et al., 2024) suggest that legume and other cover crops with a narrow C/N ratio in their tissue at termination should transfer N to the following cash crops and thus reduce the amount of fertilizer needed. However, there are still many challenges in our ability to track N in cover crop tissue as it moves through the soil–plant–water–atmosphere system; in many studies, the fate of much of the N remains unaccounted for (Lacey et al., 2020). The hydrology of nutrient loss by below‐ and aboveground water flows is also very complicated on a watershed scale, making it difficult to know exactly how N conservation practices affect downstream water quality (Blankenship, 2025). Additionally, most cropland N leaching research has focused almost exclusively on nitrate‐N losses, but our recent analyses (Owens & Weil, 2023) of groundwater, soil porewater, and surface runoff samples indicate that dissolved organic N may equal or surpass nitrate‐N loss by these pathways, and that cover crops have little effect on the non‐nitrate portion of N losses. While the eutrophication potential of organic N dissolved in stream water has been extensively studied (Feng et al., 2015; Keller & Hood, 2011), additional research is needed to assess dissolved organic N losses from croplands, and how this source of soluble N might be integrated into the Chesapeake Bay Model (Chesapeake Bay Program, 2024) that assesses and guides much of the restoration effort.

Maryland has endorsed cover crops as a key best management practice to reduce N loads to the Chesapeake Bay. Annual plantings are cited as a key goal in the MDA Phase 3 Watershed Implementation Plan and reflected accordingly in state budgets as the primary, cost‐effective practice within the agricultural sector. Increasingly, MDA sees demand to increase cover crop acres to achieve multiple environmental outcomes (e.g., water quality, soil health, and climate resiliency). Accordingly, cover crop program incentives are evaluated annually, guided by regular engagement with MDA's partnership with institutions of higher education. MDA aims to involve research to inform the program. For example, recent efforts include a partnership to employ remote sensing for verification of spring cover crop termination (Gao et al., 2023). This provides time savings for MDA staff conducting field verification, but could also provide an ability to assess and characterize biomass for soil carbon estimations. Recent developments in remote sensing now enable cost‐effective near real‐time evaluation of cover crop adoption, cover biomass production, and even cover crop N content (Hively et al., 2020; Thieme et al., 2022, 2025). These remote sensing advances are being used by MDA to obtain more information on its cover crop program impacts to aid in developing program improvements. Because these advances could also provide the capability to assess cover crop biomass and N uptake achieved, there has been some consideration of possibly converting the cover crop cost share to a pay‐for‐outcome (e.g., ground cover, biomass C and N achieved) rather than a pay‐for‐practice (planting the seeds in fall and terminating the cover crop in spring). However, such a change has not been made as it would shift the risks solely to the farmer rather than promote adoption by having society share in the risks added by cover cropping. In addition, even models that do a very good job of predicting average trends across sites can be wildly off for a given individual field, which is what an individual farmer receiving—or not receiving—a subsidy payment cares about. Yet, having at least a portion of the incentive payments based on performance rather than practice still deserves discussion within the region's overlapping environmental and agricultural community as a means of stretching limited dollars available to support cover crop adoption and getting greater environmental benefits from each dollar spent. There could be value in focusing at least part of any performance assessment on N and biomass accumulated by late‐fall, since cover crop N accumulated in spring is more likely to reduce soil N available to the subsequent crop and less likely to reflect capture of deep N that was about to leach to groundwater.

## 7 CONCLUSION

In conclusion, Maryland's history with the Chesapeake Bay led the state to become an early adopter of many approaches to the management of N that would now be considered emblematic of regenerative agriculture. Less tillage, a more rational, conservative approach to N fertilizer and manure recommendations, and widespread use of winter cover crops have made a positive difference in N‐use efficiency and reduced water quality impacts, while maintaining highly productive agriculture. Other states and regions could learn from Maryland's experience over the past half‐century. Two cornerstones of policy stand out as contributing to Maryland's success so far: (1) subsidies have been large enough to change farmers' calculations and behavior, and (2) the administration of the program has been nimble and flexible enough to react quickly to changes in the science, farmers’ practices, and even real‐time weather conditions. Nevertheless, control of the remaining N losses is a continuing challenge. With the adoption of cover cropping now widespread and relatively stable in Maryland, the next steps could focus on enhancing the management of cover crops to maximize their capture of deep soil N and its cycling in ways that could further reduce the amount and expense of N fertilizer inputs. Some portion of cover crop subsidies could be tied to satellite‐monitored late fall N accumulation, despite the caveats mentioned above. More research and funding could go toward earlier planting of cover crop mixtures, including payments to offset the cost of irrigating aerially‐intersown cover crop seed when conditions are dry, and irrigation is available. While cover crops have the potential to reduce nitrate leaching losses to near‐zero, more research is needed on other strategies that may reduce cropland losses of dissolved organic N.

## AUTHOR CONTRIBUTIONS

**Ray Weil**: Conceptualization; data curation; formal analysis; investigation; methodology; visualization; writing—original draft. **Alisha Mulkey**: Conceptualization; investigation; resources; validation; writing—review and editing.

## CONFLICT OF INTEREST STATEMENT

The authors declare no conflicts of interest.

## References

[jeq270316-bib-0001] Arrington, K. E. , Ordóñez, R. A. , Rivera‐Ocasio, Z. , Luthard, M. , Tierney, S. , Spargo, J. , Finney, D. , Kaye, J. , & White, C. (2024). Improving a nitrogen mineralization model for predicting unfertilized corn yield. Soil Science Society of America Journal, 88, 905–920. 10.1002/saj2.20665

[jeq270316-bib-0002] Bandel, V. A. , Dzienia, S. , Stanford, G. , & Legg, J. O. (1975). N behavior under no‐till vs. conventional corn culture. I. First‐year results using unlabeled N fertilizer. Agronomy Journal, 67, 782–786. 10.2134/agronj1975.00021962006700060014x

[jeq270316-bib-0003] Bandel, V. A. , & Fox, R. H. (1984). Management of nitrogen in New England and Middle Atlantic states. In R. D. Hauck (Ed.), Nitrogen in crop production (pp. 675–689–1). Wiley. 10.2134/1990.nitrogenincropproduction.c46

[jeq270316-bib-0004] Blankenship, K. (1994). Maryland nearly halfway to nutrient management goal. Bay Journal. https://www.bayjournal.com/news/pollution/maryland‐nearly‐halfway‐to‐nutrient‐management‐goal/article_80bbd943‐f298‐5c4a‐b185‐fc1e8eed8343.html

[jeq270316-bib-0005] Blankenship, K. (2002). Bay states seek federal support for pilot programs to curb ag runoff. Bay Journal: The Chesapeake Bay Newspaper, 12, 6.

[jeq270316-bib-0006] Blankenship, K. (2025). Study aims for reality check on nutrient runoff from farms. Bay Journal. https://www.bayjournal.com/news/study‐aims‐for‐reality‐check‐on‐nutrient‐runoff‐from‐farms/article_1d9b2db6‐7b80‐439e‐b96a‐04e61f498655.html

[jeq270316-bib-0007] Brainard, J. (2014). Trading away toward a cleaner Bay. Chesapeake Quarterly, 13, 1–4. https://www.chesapeakequarterly.net/V13N1/main1/

[jeq270316-bib-0008] Chesapeake Bay Program . (2024). Chesapeake assessment and scenario tool (cast) version 2023, phase 6 . https://cast.chesapeakebay.net/documentation/modeldocumentation

[jeq270316-bib-0009] Dean, J. E. , & Weil, R. R. (2009). Brassica cover crops for nitrogen retention in the Mid‐Atlantic coastal plain. Journal of Environmental Quality, 38, 520–528. http://jeq.scijournals.org/cgi/content/abstract/joenq;38/2/520 10.2134/jeq2008.0066 19202022

[jeq270316-bib-0010] Feng, Y. , Friedrichs, M. A. M. , Wilkin, J. , Tian, H. , Yang, Q. , Hofmann, E. E. , Wiggert, J. D. , & Hood, R. R. (2015). Chesapeake Bay nitrogen fluxes derived from a land‐estuarine ocean biogeochemical modeling system: Model description, evaluation, and nitrogen budgets. Journal of Geophysical Research: Biogeosciences, 120, 1666–1695. 10.1002/2015JG002931 PMC5014239 27668137

[jeq270316-bib-0011] Frankel, L. T. , Friedrichs, M. A. M. , St‐Laurent, P. , Bever, A. J. , Lipcius, R. N. , Bhatt, G. , & Shenk, G. W. (2022). Nitrogen reductions have decreased hypoxia in the Chesapeake Bay: Evidence from empirical and numerical modeling. Science of the Total Environment, 814, 152722. 10.1016/j.scitotenv.2021.152722 https://www.sciencedirect.com/science/article/pii/S0048969721078013 34974013

[jeq270316-bib-0012] Gao, F. , Jennewein, J. , Hively, W. D. , Soroka, A. , Thieme, A. , Bradley, D. , Keppler, J. , Mirsky, S. , & Akumaga, U. (2023). Near real‐time detection of winter cover crop termination using harmonized landsat and sentinel‐2 (hls) to support ecosystem assessment. Science of Remote Sensing, 7, 100073. 10.1016/j.srs.2022.100073, https://www.sciencedirect.com/science/article/pii/S2666017222000359

[jeq270316-bib-0013] Gentry, L. E. , Green, J. M. , Mitchell, C. A. , Andino, L. F. , Rolf, M. K. , Schaefer, D. , & Nafziger, E. D. (2024). Split fertilizer nitrogen application with a cereal rye cover crop reduces tile nitrate loads in a corn–soybean rotation. Journal of Environmental Quality, 53, 90–100. 10.1002/jeq2.20530 37940131

[jeq270316-bib-0014] Hadas, A. , & Portnoy, R. (1994). Nitrogen and carbon mineralization rates of composted manures incubated in soil. Journal of Environmental Quality, 23, 1184–1189. 10.2134/jeq1994.00472425002300060008x https://www.soils.org/publications/jeq/abstracts/23/6/1184

[jeq270316-bib-0015] Hansen, D. J. (2006). Manure as a nutrient source. In K. C. Haering & G. K. Evanylo (Eds.), The mid‐Atlantic nutrient management handbook (pp. 208–225). Mid‐Atlantic Regional Water Program.

[jeq270316-bib-0016] Hirsh, S. (2018). Deep soil nitrogen capture and recycling by early‐planted, deep‐rooted cover crops [Doctoral dissertation, University of Maryland]. DRUM. https://drum.lib.umd.edu/handle/1903/21318

[jeq270316-bib-0017] Hirsh, S. M. , Duiker, S. W. , Graybill, J. , Nichols, K. , & Weil, R. R. (2021). Scavenging and recycling deep soil nitrogen using cover crops on mid‐Atlantic, USA farms. Agriculture, Ecosystems & Environment, 309, 107274. 10.1016/j.agee.2020.107274

[jeq270316-bib-0018] Hirsh, S. M. , & Weil, R. R. (2019). Deep soil cores reveal large end‐of‐season residual mineral nitrogen pool. Agricultural & Environmental Letters, 4, 180055. 10.2134/ael2018.10.0055

[jeq270316-bib-0019] Hively, W. D. , Lee, S. , Sadeghi, A. M. , McCarty, G. W. , Lamb, B. T. , Soroka, A. , Keppler, J. , Yeo, I.‐Y. , & Moglen, G. E. (2020). Estimating the effect of winter cover crops on nitrogen leaching using cost‐share enrollment data, satellite remote sensing, and soil and water assessment tool (SWAT) modeling. Journal of Soil and Water Conservation, 75, 362–375. 10.2489/jswc.75.3.362

[jeq270316-bib-0020] Keller, D. P. , & Hood, R. R. (2011). Modeling the seasonal autochthonous sources of dissolved organic carbon and nitrogen in the upper Chesapeake Bay. Ecological Modelling, 222, 1139–1162. 10.1016/j.ecolmodel.2010.12.014. https://www.sciencedirect.com/science/article/pii/S0304380010006800

[jeq270316-bib-0021] Lacey, C. , Nevins, C. , Camberato, J. , Kladivko, E. , Sadeghpour, A. , & Armstrong, S. (2020). Carbon and nitrogen release from cover crop residues and implications for cropping systems management. Journal of Soil and Water Conservation, 75(4), 505–514. 10.2489/jswc.2020.00102

[jeq270316-bib-0022] Lamond, R. E. , Whitney, D. A. , Hickman, J. S. , & Bonczkowski, L. C. (1991). Nitrogen rate and placement for grain sorghum production in no‐tillage systems. Journal of Production Agriculture, 4, 531–535. 10.2134/jpa1991.0531

[jeq270316-bib-0023] Magdoff, F. , Ross, D. , & Amadon, J. F. (1984). A soil test for nitrogen availability to corn. Soil Science Society of America Journal, 48, 1301–1304.

[jeq270316-bib-0024] Magette, W. L. , Weismiller, R. A. , Angle, J. S. , & Brinsfield, R. B. (1989). A nitrate groundwater standard for the 1990 farm bill. Journal of Soil and Water Conservation, 44, 491–494. http://www.jswconline.org/content/44/5/491.abstract

[jeq270316-bib-0025] Marcillo, G. S. , Thapa, R. , Mirsky, S. B. , & Martin, N. (2024). The nitrogen value of cover crops: How much N can cover crops replace?. Agricultural & Environmental Letters, 9, e70006. 10.1002/ael2.70006

[jeq270316-bib-0026] Maryland Department of Environment . (2025). Maryland's phase iii watershed implementation plan (WIP). Maryland Department of the Environment. https://mde.maryland.gov/programs/water/TMDL/TMDLImplementation/Pages/Phase3WIP.aspx

[jeq270316-bib-0027] Meisinger, J. J. , Bandel, V. A. , Angle, J. S. , O'Keefe, B. E. , & Reynolds, C. M. (1992). Presidedress soil nitrate test evaluation in Maryland. Soil Science Society of America Journal, 56, 1527–1532. 10.2136/sssaj1992.03615995005600050032x

[jeq270316-bib-0028] Morris, T. F. , Murrell, T. S. , Beegle, D. B. , Camberato, J. J. , Ferguson, R. B. , Grove, J. , Ketterings, Q. , Kyveryga, P. M. , Laboski, C. A. M. , McGrath, J. M. , Meisinger, J. J. , Melkonian, J. , Moebius‐Clune, B. N. , Nafziger, E. D. , Osmond, D. , Sawyer, J. E. , Scharf, P. C. , Smith, W. , Spargo, J. T. , … Yang, H. (2017). Strengths and limitations of nitrogen rate recommendations for corn and opportunities for improvement. Agronomy Journal, 110(1), 1–37. 10.2134/agronj2017.02.0112

[jeq270316-bib-0029] Owens, V. , & Weil, R. (2023). Groundwater chemistry under enhanced and standard cover crop treatments in a commodity grain field. American Society of Agronomy and the Soil Science Society of America. https://scisoc.confex.com/scisoc/2023am/meetingapp.cgi/Paper/154248

[jeq270316-bib-0030] Preusch, P. L. , Adler, P. R. , Sikora, L. J. , & Tworkoski, T. J. (2002). Nitrogen and phosphorus availability in composted and uncomposted poultry litter. Journal of Environmental Quality, 31, 2051–2057. http://jeq.scijournals.org/cgi/content/abstract/31/6/2051 10.2134/jeq2002.2051 12469856

[jeq270316-bib-0031] Quinn, D. J. , Poffenbarger, H. J. , Leuthold, S. J. , & Lee, C. D. (2021). Corn response to in‐furrow fertilizer and fungicide across rye cover crop termination timings. Agronomy Journal, 113, 3384–3398. 10.1002/agj2.20723

[jeq270316-bib-0032] Quinn, D. J. , Poffenbarger, H. J. , Miguez, F. E. , & Lee, C. D. (2023). Corn optimum nitrogen fertilizer rate and application timing when following a rye cover crop. Field Crops Research, 291, 108794. 10.1016/j.fcr.2022.108794 https://www.sciencedirect.com/science/article/pii/S0378429022003653

[jeq270316-bib-0033] Ruffatti, M. D. , Roth, R. T. , Lacey, C. G. , & Armstrong, S. D. (2019). Impacts of nitrogen application timing and cover crop inclusion on subsurface drainage water quality. Agricultural Water Management, 211, 81–88. 10.1016/j.agwat.2018.09.016 http://www.sciencedirect.com/science/article/pii/S0378377418313738

[jeq270316-bib-0034] Schulte, D. M. (2017). History of the Virginia oyster fishery, Chesapeake Bay, USA. Frontiers in Marine Science, 4, 1–19. 10.3389/fmars.2017.00127

[jeq270316-bib-0035] Scott, D. A. , Johnson, J. M. F. , & Gesch, R. W. (2022). Cover crop and nitrogen rate management practices influence corn NDVI and nitrogen content. Agronomy Journal, 114, 2473–2483. 10.1002/agj2.21085

[jeq270316-bib-0036] Sedghi, N. , Fox, R. J. , Sherman, L. , Gaudlip, C. , & Weil, R. R. (2023). Aerial interseeding and planting green to enhance nitrogen capture and cover crop biomass carbon. Journal of Soil and Water Conservation, 78(3), 282–298. 10.2489/jswc.2023.00051

[jeq270316-bib-0037] Sedghi, N. , & Weil, R. (2022). Fall cover crop nitrogen uptake drives reductions in winter‐spring leaching. Journal of Environmental Quality, 51, 337–351. 10.1002/jeq2.20342 35290665

[jeq270316-bib-0038] Staver, K. W. , & Brinsfield, R. B. (1998). Using cereal grain winter cover crops to reduce groundwater nitrate contamination in the mid‐Atlantic coastal plain. Journal of Soil and Water Conservation, 53, 230–240.

[jeq270316-bib-0039] Swinker, M. , Tester, P. , Attix, D. K. , & Schmechel, D. (2002). Human health effects of exposure to Pfiesteria piscicida: A review. Microbes and Infection, 4, 751–762. 10.1016/S1286-4579(02)01594-0 12067835

[jeq270316-bib-0040] Terlizzi, D. (2006). *Pfiesteria* hysteria, agriculture, and water quality in the Chesapeake Bay: The extension bridge over troubled waters. Journal of Extension, 44, Article 5. https://tigerprints.clemson.edu/joe/vol44/iss5/5

[jeq270316-bib-0041] Thieme, A. , Hively, W. D. , Gao, F. , Jennewein, J. , Mirsky, S. , Soroka, A. , Keppler, J. , Bradley, D. , Skakun, S. , & McCarty, G. W. (2022). Remote sensing evaluation of winter cover crop springtime performance and the impact of delayed termination. Agronomy Journal, 115, 42–458. 10.1002/agj2.21207

[jeq270316-bib-0042] Thieme, A. , Jennewein, J. , Hively, W. D. , Lamb, B. T. , Whitcraft, A. K. , Mirsky, S. B. , Reberg‐Horton, S. C. , & Justice, C. (2025). Multispectral red‐edge indices accurately estimate nitrogen content in winter cereal cover crops. Agronomy Journal, 117, e70011. 10.1002/agj2.70011

[jeq270316-bib-0043] USDA/NASS . (2024). National agricultural statistics service quick stats. United States Department of Agriculture. https://quickstats.nass.usda.gov/

[jeq270316-bib-0044] Wang, F. , & Weil, R. R. (2018). The form and vertical distribution of soil nitrogen as affected by forage radish cover crop and residual side‐dressed N fertilizer. Soil Science, 183, 22–33. 10.1097/ss.0000000000000224

[jeq270316-bib-0045] Waring, E. R. , Lagzdins, A. , Pederson, C. , & Helmers, M. J. (2020). Influence of no‐till and a winter rye cover crop on nitrate losses from tile‐drained row‐crop agriculture in Iowa. Journal of Environmental Quality, 49, 292–303. 10.1002/jeq2.20056 33016415

[jeq270316-bib-0046] Weil, R. R. , Weismiller, R. A. , & Turner, R. S. (1990). Nitrate contamination of groundwater under irrigated coastal plain soils. Journal of Environmental Quality, 19, 441–448. 10.2134/jeq1990.00472425001900030015x

[jeq270316-bib-0047] White, C. M. , Sigdel, S. , Karsten, H. D. , Meinen, R. J. , & Spargo, J. T. (2023). Recalibrating the Pennsylvania pre‐sidedress soil nitrate test recommendations for modern corn production. Agronomy Journal, 115(5), 2600–2613. 10.1002/agj2.21426

[jeq270316-bib-0048] Zou, T. , Davidson, E. A. , Sabo, R. D. , MacDonald, G. K. , & Zhang, X. (2024). Disparities in nitrogen and phosphorus management across time and space: A case study of the Chesapeake Bay using the CAFE framework. Environmental Research Letters, 19, 114016. 10.1088/1748-9326/ad786c PMC11977706 40207068

